# Evaluation of KU-F40 automated microscope for parasitology: when artificial intelligence meets old school microscopy

**DOI:** 10.1128/jcm.00743-25

**Published:** 2026-02-10

**Authors:** Antoine Aupaix, Lorenzo Filippin, Justine Jaumot, Stéphanie Cannoot, Monia Chemais, Delphine Martiny, Véronique Yvette Miendje Deyi, Marine Deffontaine, Corentin Deckers, Valérie Verbelen, Idzi Potters, Charlotte Drieghe, Samy Mzougui, Reza Soleimani, Patrick Philippart, Jonathan Brauner

**Affiliations:** 1 Centre Hospitalier EpiCURA, Hornu 218727 , Hornu, Belgium; 2 Centre Hospitalier Regional Haute Senne, Soignies, Belgium; 3 Department of Microbiology, Laboratoire Hospitalier Universitaire de Bruxelles-Brussel Universitair Laboratorium (LHUB-ULB), Université Libre de Bruxelles 26659, Brussels, Belgium; 4 Faculty of Medicine and Pharmacy, University of Mons (UMONS) 82383, Mons, Belgium; 5 Center Hospitalier Mouscron 82388, Mouscron, Belgium; 6 Centre Hospitalier Universitaire UCL Namur site Mont-Godinne 82470 , Yvoir, Belgium; 7 Cliniques Saint-Pierre Ottignies 82408 , Ottignies-Louvain-la-Neuve, Belgium; 8 Department of Clinical Sciences, Institute of Tropical Medicine 567788 , Antwerp, Belgium; 9 Centre Hospitalier Universitaire de Liège 37472, Liège, Belgium; 10 Centre Hospitalier Universitaire Ambroise Paré 82241 , Mons, Belgium; Mayo Clinic Minnesota, Rochester, Minnesota, USA

**Keywords:** parasites, intestinal parasitic infection, automation, microscopy

## Abstract

Intestinal parasitic infections (IPIs) have a worldwide distribution and have a major impact on health, work capacity, and economy in many countries. Light microscopy is still considered the reference method for IPI diagnosis but is labor-intensive. KU-F40, an automated feces analyzer, combines automated microscopic examination of stool samples and deep learning artificial intelligence. The aim of this study is to evaluate the performance of KU-F40 for the diagnosis of IPI. A random collection of stool samples prescribed for IPI investigation was retrospectively collected from six clinical laboratories in Belgium along with external quality controls. All samples were analyzed in our laboratory by wet mount preparation using classic light microscopy as reference. We assessed the sensitivity and specificity for parasite detection/identification. Finally, we studied the improvement in parasite detection rate when increasing the number of pictures taken to 150% and 200% of the standard settings. A total of 267 clinical stool samples were included. Using standard settings, overall sensitivity and specificity were 86% and 45%, respectively. When considering only clinically relevant parasites, sensitivity was 95%. Increasing the number of pictures allowed to improve detection rate, but it remained under 90% for several targets. KU-F40 offers an innovative approach and provides welcome automation in the diagnosis of IPI. Currently, its performance does not allow it to be used as a screening tool with automatic validation of negative results. Critical missing features could enhance its performance, including the addition of a 10x magnification objective and additional parasites currently absent from the database.

**IMPORTANCE:**

Intestinal parasitic infections have a worldwide distribution and are a global health concern in many countries. Light microscopy is still considered the reference method for diagnosis but is labor-intensive, time-consuming, and requires highly skilled and motivated technologists. In this paper, we evaluate the KU-F40, an automated feces analyzer designed to diagnose intestinal parasitic infections by combining automated light microscopy and deep learning artificial intelligence for detection and presumptive identification of several protozoans and helminths. As it relies on microscopy, this method enables the detection and identification of a predefined panel of parasites, whose morphology is known to the system and included in the database, without requiring prior diagnostic suspicion, similarly to multiplex PCR assays. The automation could improve the quality, standardization, and turnaround time of stool parasitology. This study is the first to evaluate the performance of the KU-F40 on a wide range of parasites, collected from six Belgian hospitals, including our two national reference centers.

## INTRODUCTION

Intestinal parasitic infections (IPIs) are worldwide distributed. Mortality rates are relatively low, as opposed to vector-borne disease such as malaria, but the impact on health, economy, work capacity, growth rate, and cognitive development is a real issue in many countries. Some of these are considered to be neglected tropical diseases given the limited investment allocated to research (1–3).

In Europe, IPI prevalence is low, and the most often encountered intestinal parasites are *Blastocystis hominis*, *Dientamoeba fragilis,* and commensal amoeba. Among the well-recognized pathogenic parasites, one can count *Enterobius vermicularis*, *Giardia duodenalis,* and *Cryptosporidium* spp. (1, 4) Diagnostic management is particularly challenging for IPI for multiple reasons: low or intermittent parasite excretion, difficulties in preserving parasites in stool samples, the need for multiple samples to achieve a good sensitivity with microscopy. Identification with light microscopy is still considered the reference method, but it is very labor-intensive and time-consuming (5, 6). It requires ideally three samples from consecutive days and highly skilled technologists to achieve a sensitivity greater than 90%. This is difficult to achieve in clinical laboratories due to a lack of technicians with microscope proficiency and adequate training (7).

Other methods have been developed to improve IPI diagnostics. Enzymatic immunoassays and immunochromatographic assays are particularly easy to use. The analytical performance can vary depending on the pathogen targeted and the manufacturer (8).

Molecular assays have become a key feature in IPI diagnosis in an increasing number of laboratories. Sensitivity of commercial qPCR is generally excellent and is often superior to light microscopy especially for the most encountered pathogenic protozoans (*G. duodenalis, Cryptosporidium* spp., and *E. histolytica*). The sensitivity for helminths is less convincing, notably for *Enterobius vermicularis*, *Ascaris lumbricoides*, *Trichuris trichiura*, *Strongyloides stercoralis,* and the hookworms (9–12). Another drawback that molecular tests have is their need for diagnostic suspicion as they target a limited set of pathogens.

Recently, several systems using automated microscopy have become available on the market. This study evaluates the KU-F40 (Zhuhai Keyu Biological Engineering Co., Ltd. Guangdong, China), a fully automated feces analyzer that allows microscopic examination of fresh or fixated stool samples. A deep learning artificial intelligence (AI) recognition function allows for detection and presumptive identification of parasites found. Those analyzers also provide multiple lateral flow antigen tests with full automation (sample preparation and loading into the well, test readout and image archiving) that may be ordered with or without microscopic examination of each specimen.

This study aimed at assessing analytical performance of the KU-F40 automated microscope for IPI diagnosis in comparison to the reference method, i.e., manual light microscopy on a collection of negative and positive samples retrieved from our routine work, five other clinical laboratories, and external quality controls.

## MATERIALS AND METHODS

### Study area

This study was conducted at the clinical laboratory of EpiCURA Hospital Center, an 806-bed secondary-care facility serving the Mons-Borinage and Ath regions of Belgium.

### Biological samples

We selected clinical samples, with or without fixatives (including SAF and formalin 10%) that requested microscopic examination for parasites. Retrieved between April and September 2024, they originated from routine clinical samples from our home institution as well as from five other clinical laboratories in Belgium: Laboratoire Hospitalier Universitaire de Bruxelles-Universitair laboratorium Brussel (LHUB-ULB), Institute of Tropical Medicine Antwerp, Centre Hospitalier Régional Haute Senne Soignies, Clinique St-Pierre Ottignies, and Centre Hospitalier de Mouscron. In addition, external quality controls from Sciensano (previously the National Public Health Institute) and UK NEQAS scheme for microbiology (UK NEQAS, Sheffield, United Kingdom) were also tested. Helminth and protozoan diagnoses were first conducted on microscopic examination.

All samples were analyzed in our laboratory with light microscopy by trained technologists. Prior to analysis with both methods, all samples underwent a concentration technique using SAF fixative with dual vertical mesh filtration and sedimentation, processed in a closed system (Mini Parasep SF, Apacor Ltd., Wokingham, United Kingdom). From each sample, a small amount was mixed with a few drops of saline, stained with 10 µL of Kop-Color (ELIstain Para-Color from ELITechGroup, Puteaux, France) on an object-carrying slide and examined under a 22 × 22 mm coverslip. Kop-Color reagent, composed of Lugol and specific dyes, enhanced contrast between parasitic elements (stained yellow to brown) and the blue-colored background. The entire coverslip is examined with the 10× objective, then approximately 300 fields with the 40× objective. When clinical data or microscopic appearance suggest *Cyclospora, Cystoisospora,* or *Cryptosporidium,* the sample is examined with fluorescence microscopy after auramine-phenol stain as well as without stain to look for auto-fluorescence. All samples, internal or external, were examined the same day that the analysis was conducted with KU-F40.

### KU-F40 – automated microscopy

After concentration, the sample is transferred into the main chamber of the sample collection cup (samples with or without fixative: 0.5 to 1.0 g of solid stool, 0.5 mL of watery stool). The machine first takes a photo of the stool sample to assess consistency and then adds 0.75 mL of diluent. Homogenization is achieved by a rotative spoon fixed in the cap, and the sample then migrates into a smaller “analysis chamber,” separated from the main chamber by a filter with 320 µm pores.

**Fig 1:**
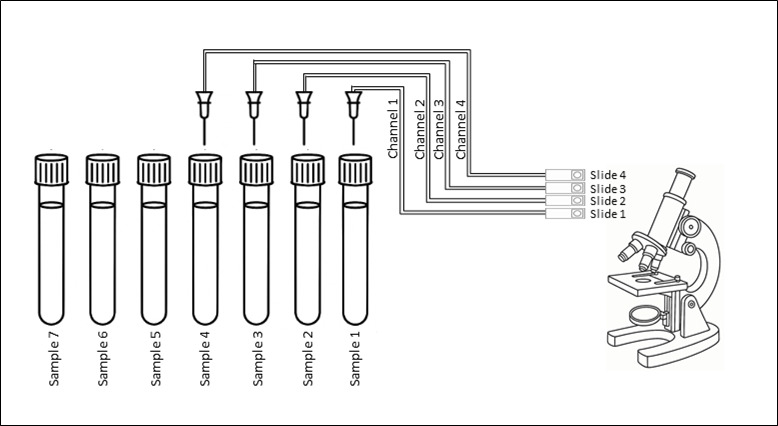
Schematic representation of internal components of KU-F40, showing the integrated microscope and the four different channels, consisting of four individual aspiration needles, each connected to dedicated tubing, delivering the sample onto one of four slides for examination. In this example, channel 1 will process sample 1 then 5, channel 2 will process sample 2 then 6, etc. The microscope sequentially reads slides 1 through 4.

Fifty microliters was then taken for parasitological analysis. This volume is injected into one of the four channels and spread over one of the four slides available for analysis. Each channel/slide is sequentially used as samples are processed, ensuring redundancy in the event of a malfunction (see Fig. 1). An integrated optical microscope then takes 400 images at objective ×20 (corresponding to 50 fields at objective ×20 of a conventional microscope) and 60 pictures at objective ×40 (corresponding to 20 fields at ×40 of a conventional microscope). This number of pictures is the default setting in the standard mode of examination and can be increased to 768 pictures at ×20 and 120 at ×40. Other examination modes are available, including iodine staining to differentiate better among amoeba species (0.3 mL of iodine stain is added to the sample, in addition to the diluent). In this case, the samples must be reanalyzed after selecting the iodine staining mode beforehand.

All the pictures taken are then analyzed by an AI that compares them to a series of parasites present in its database (Table 1). Other items can be automatically flagged, such as red and white blood cells, Charcot-Leyden crystals, plant cells, muscle fibers, fungus, epithelial cells, starch granules, etc. Those items, provided by the manufacturer, can be manually disabled as some are irrelevant for parasitology. When a potential parasite form is found, identification is triggered if the recognition score assigned by the AI is higher than the identification threshold previously set for each parasite searched for (see the supplementary material at https://doi.org/10.5281/zenodo.18268350). This score is based on the visual characteristics of the shape analyzed. All the images can then be viewed, with those considered positive ranked first. Although users can submit images to the R&D department to contribute to the database, the system operates under version control. As such, a version update is required before any modifications can be implemented.

**TABLE 1:** Parasites as listed in the KU-F40 database, with automatic detection and identification

Helminths	Protozoans
Liver fluke egg^*a*^	*Endolimax nana* cysts
*Ascaris* egg	*Entamoeba histolytica* cysts^*c*^
Hookworm egg	*Entamoeba coli* cysts
Pinworm egg	*Giardia duodenalis* cysts
Whipworm egg	*Blastocystis hominis* cysts
Tapeworm egg	
*Diphyllobothrium^b^* egg	
*Strongyloides stercoralis* larvae	

^*a*^ *Opisthorchidae/Heterophiidae* are identified as “liver fluke” in KU-F40 database.

^*b*^ *Adenocephalus/Dibothriocephalus/Diphyllobothrium *spp*. *are listed as “*Diphyllobothrium”*.

^*c*^ The species *histolytica* is listed but the KU-F40 cannot differentiate with *Entamoeba dispar*.

### Data analysis

#### Accuracy

Accuracy is defined as the degree of closeness of a measured value to a standard. External quality controls for parasitology challenge, from UK NEQAS (United Kingdom) scheme and Sciensano (Belgium) were processed by the KU-F40 as clinical specimens to assess accuracy (concordance between detection/identification provided and the expected result).

#### Contamination study

We selected one sample exhibiting a high burden of *Giardia duodenalis* cysts, a frequently encountered pathogen, resistant to ozonolysis and chlorination. This sample was analyzed four times, alternating with a tube of physiological saline as a negative control, to assess any inter-sample contamination.

#### Comparison study

Clinical samples were analyzed on the same day with the KU-F40 with standard settings (400 images at ×20, 60 images at ×40 magnification) and compared with manual light microscopy as a reference. Sensitivity, specificity, and false positive and negative rates were calculated. Here, the sensitivity was defined as the per-sample sensitivity, calculated as the number of true positive samples detected by KU-F40 (regardless of whether the sample was mono- or multi-parasitic) divided by all positive samples with the reference method. All samples detected as positive with our reference method were then re-analyzed on the KU-F40 by increasing the number of pictures to 150% (600 images at ×20, 90 at ×40 magnification), then to 200% (768 at ×20 and 120 at ×40). The changes in detection rate or per-pathogen sensitivity, defined as the total of parasites detected by KU-F40 divided by the total of parasites present across all samples, were recorded. We also evaluated the rate of correct identification for the parasites presented in the AI database, specifically whether the genus (and eventually species if specified) provided by the KU-F40 are consistent with manual microscopy. Finally, to assess whether the discrepancies were due to a lack of camera sensitivity (absence of parasites in the images) or a failure of the AI to recognize the parasites (parasites present in the images but not detected), we systematically reviewed all captured images.

#### Turnaround time

The time needed to perform the analysis, i.e., pre-treatment including centrifugation, removal of eluate and manual microscopy for the reference method, and transfer of sample into the dedicated tubes, automated microscopic analysis and review of positive samples before validation for KU-F40, was measured on three different batches of 20 samples. Three distinct technologists carried out these analyses by pairs, on three different days, with one technologist performing the standard microscopy and a second technologist processing the same samples with the KU-F40.

## RESULTS

### Analytical performance of KU-F40

Results for accuracy using external quality controls are shown in Table 2. Our contamination study showed no contamination with *G. duodenalis* cysts between samples. However, as shown below, we did find a case of inter-sample contamination in the comparison study with an egg of *Taenia* spp.

**TABLE 2:** Accuracy of KU-F40 with external quality controls

Sample number	Species included	Detection	Misidentification
P19509	*Giardia duodenalis*	Yes	*Blastocystis hominis^a^*
P19733	*Giardia duodenalis*	Yes	
P19997	*Enterobius vermicularis*	Yes	
P20274	*Ancylostomidae*	Yes	
	*Giardia duodenalis*	Yes	
	*Endolimax nana*	Yes	
P20794	*Enterobius vermicularis*	Yes	
P20795	*Trichuris trichiura*	No	
UKNEQAS8716	*Trichostrongylus*	Yes	
	*Entamoeba histolytica/E. dispar*	Yes	
UKNEQAS8717	*Ancylostoma duodenale*	Yes	
Total	Out of the 8 samples	88% (7/8)	
	Out of the 11 parasites	91% (10/11)	

^*a*^ The sample was correctly flagged for *Giardia duodenalis*, but some cysts were mistaken for *Blastocystis hominis*.

### Comparison study

A total of 267 stool samples were included, including 80 positive samples with 111 different parasites. Details on the samples and parasites included are shown in Table 3.

**TABLE 3:** Samples and parasites included in the comparison study

	*N*	Percentage
Samples		
Negative	187	70%
Positive	80	30%
Mono-parasitic	60	23%
Multi-parasitic	20	7%
With fixative	247	93%
Without fixative	20	7%
Total	267	100%
Parasites		
Species		
Protozoans (cysts)	75	68%
*Blastocystis hominis*	15	14%
*Endolimax nana*	2	2%
*Entamoeba coli*	16	14%
*Entamoeba histolytica/E. dispar*	4	4%
*Giardia duodenalis*	25	23%
Protozoans absent from the database		
*Cyclospora* spp.	2	2%
Other amoeba^*a*^	4	4%
Other flagellates^*b*^	7	6%
Helminths	36	32%
Hookworms	2	2%
*Ascaris lumbricoides*	5	5%
*Opisthorchidae/Heterophiidae*	1	1%
*Enterobius vermicularis*	5	5%
*Strongyloides stercoralis*	3	3%
*Taenia spp*	8	7%
*Trichuris trichiura*	4	4%
Helminths absent from the database		
*Hymenolepis diminuta/Rodentolepis nana*	4	4%
*Schistosoma mansoni*	4	4%
Total	111	100%

^*a*^ Including *Iodamoeba buetschlii* and *Entamoeba hartmanni*.

^*b*^ Including *Chilomastix mesnilii* and *Dientamoeba fragilis*.

**Fig 2:**
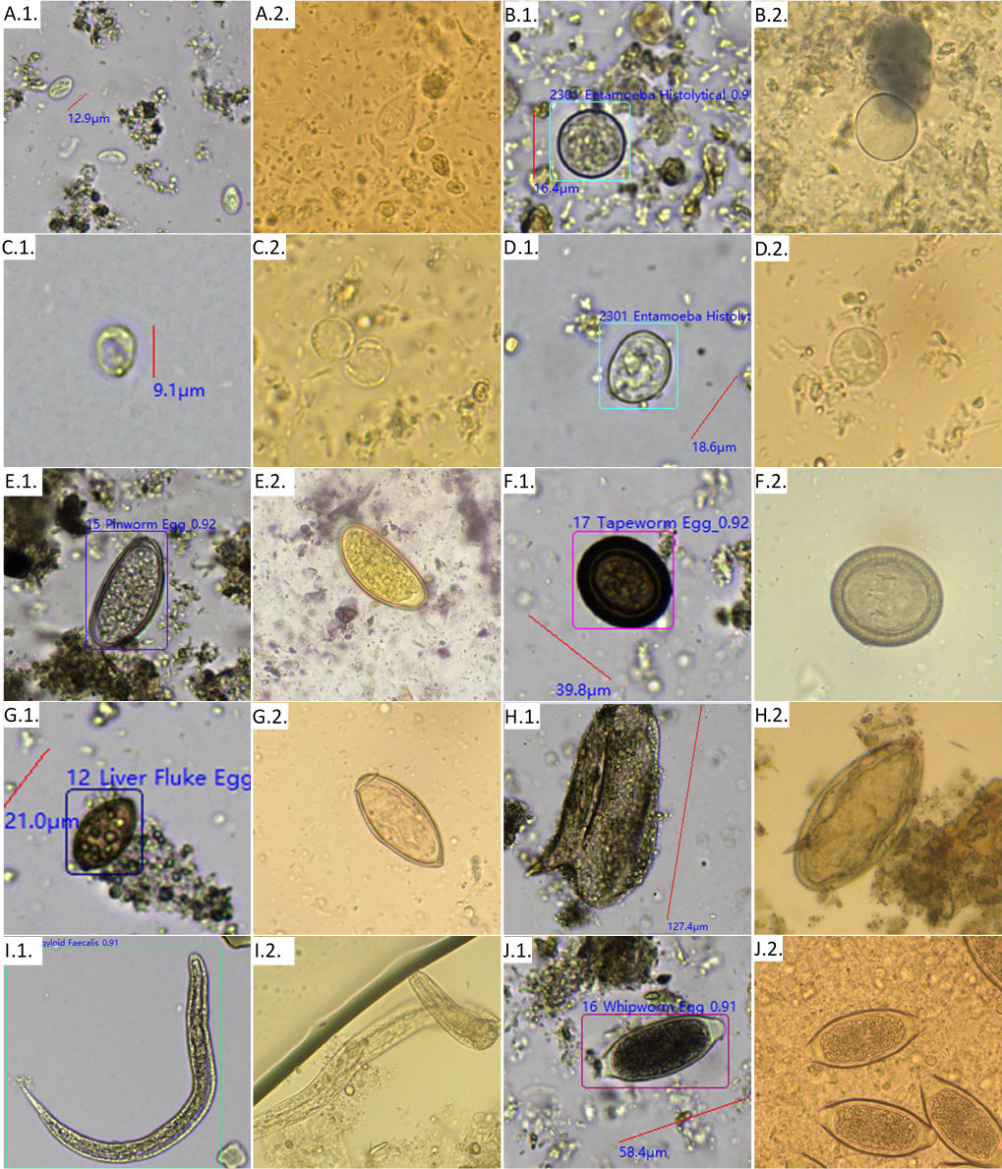
Images at 40× magnification of *Giardia duodenalis* cysts with KU-F40 (**A.1.**) and trophozoites with microscopy (**A.2.**); *Entamoeba coli* cysts with KU-F40 (**B.1.**) and microscopy (**B.2.**); *Blastocystis hominis* cysts with KU-F40 (**C.1.**) and microscopy (**C.2.**); *Entamoeba histolytica*/*dispar* cysts with KU-F40 (**D.1.**) and microscopy (**D.2.**). Images at 20x magnification of Enterobius vermicularis eggs with KU-F40 (**E.1.**) and microscopy (**E.2.**); *Taenia* spp. eggs with KU-F40 (**F.1.**) and microscopy (**F.2**.); *Opisthorchidae*/*Heterophiidae* eggs with KU-F40 (**G**.1.) and microscopy (**G.2.**); *Schistosoma mansoni* eggs with KU-F40 (**H.1.**) and microscopy (**H.2.**); *Strongyloides stercoralis* rhabditiform larva with KU-F40 (**I.1.**) and microscopy (**I.2.**); and *Trichuris trichiura* eggs with KU-F40 (**J.1.**) and microscopy (**J.2.**).

**Fig 3:**
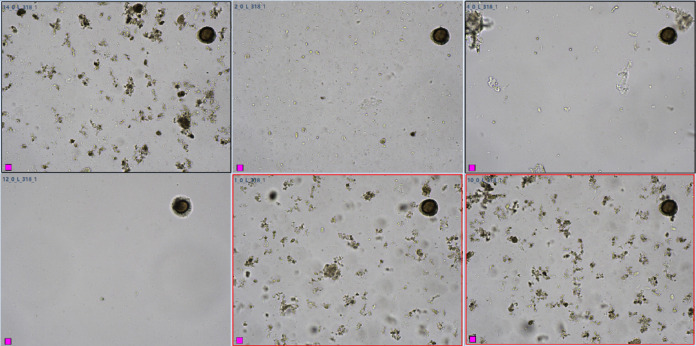
Images of one sample positive for *Taenia* spp. Eggs (top left picture) and five subsequent negative samples contaminated with the same egg on the same field L_318_1.

Data on sensitivity and specificity using the standard settings are shown in Table 4. The highest false positive rates were found with *Adenocephalus/Dibothriocephalus/Diphyllobothrium* spp. eggs (formerly *Diphyllobothrium* spp.), *Blastocystis hominis* and *Strongyloides stercoralis* larvae with rates of 10% (27/267), 10% (26/252), and 8% (22/264), respectively. Highest false negative rates were found with *Cyclospora* spp., *Entamoeba coli,* and *Schistosoma mansoni* with rates of 100% (2/2), 75% (12/16), and 75% (3/4), respectively. Figure 2 displays a panel of images obtained by KU-F40 and manual microscopy in comparison. In this comparison study, we encountered one case of contamination due to an egg of *Taenia* spp. This egg, which had adhered to the surface of one of the four slides, persisted despite inter-sample washing, resulting in the same positive image for *Taenia* spp. (i.e., field n° L_318_1 as shown in Fig. 3) for the subsequent samples. This apparent inter-sample contamination was actually due to a contaminated microscopic field, with the same egg appearing in one out of every four samples. Those contaminated samples were re-analyzed after the intervention of technicians from the company.

**TABLE 4:** Sensitivity and specificity of KU-F40, standard settings^*a*^

Samples with following parasites	Se	Sp
Protozoans	82%	80%
*Blastocystis* spp. (*N* = 15)	40%	90%
*Endolimax nana* (*N* = 2)	100%	100%
*Entamoeba coli* (*N* =16)	25%	100%
*Entamoeba histolytica/E. dispar* (*N* = 4)	75%	99%
*Giardia duodenalis* (*N* = 25)	92%	94%
Protozoans absent from database		
*Cyclospora* spp. (*N* = 2)	0%	100%
Other amoeba (*N* = 4)	50%	100%
Other flagellates (*N* = 7)	57%	100%
Helminths	89%	76%
Hookworms (*N* = 2)	50%	97%
*Ascaris lumbricoides* (*N* = 5)	80%	95%
*Opisthorchidae/Heterophiidae* (*N* = 1)	100%	99%
*Enterobius vermicularis* (*N* = 5)	100%	99%
*Strongyloides stercoralis* (*N* = 3)	67%	92%
*Taenia* spp (*N* = 8)	100%	98%
*Trichuris trichiura* (*N* = 4)	100%	98%
Helminths absent from database		
*Schistosoma mansoni* (*N* = 4)	25%	100%
*Hymenolepis diminuta/Rodentolepis nana* (*N* = 4)	50%	100%
Parasites included in database (*N* = 64)	92%	50%
Clinically relevant parasites^*b*^ (*N* = 60)	95%	74%
Total (*N* = 80)	86%	45%

^*a*^ Se, sensitivity; Sp, specificity.

^*b*^ We excluded *Entamoeba coli*, *Endolimax nana*, *Iodamoeba buetschlii*, *Entamoeba hartmanni, Chilomastix mesnilii*, *Dientamoeba fragilis, *and *Blastocystis hominis* (13).

#### Impact of the number of images on the detection rate and camera sensitivity

**Fig 4:**
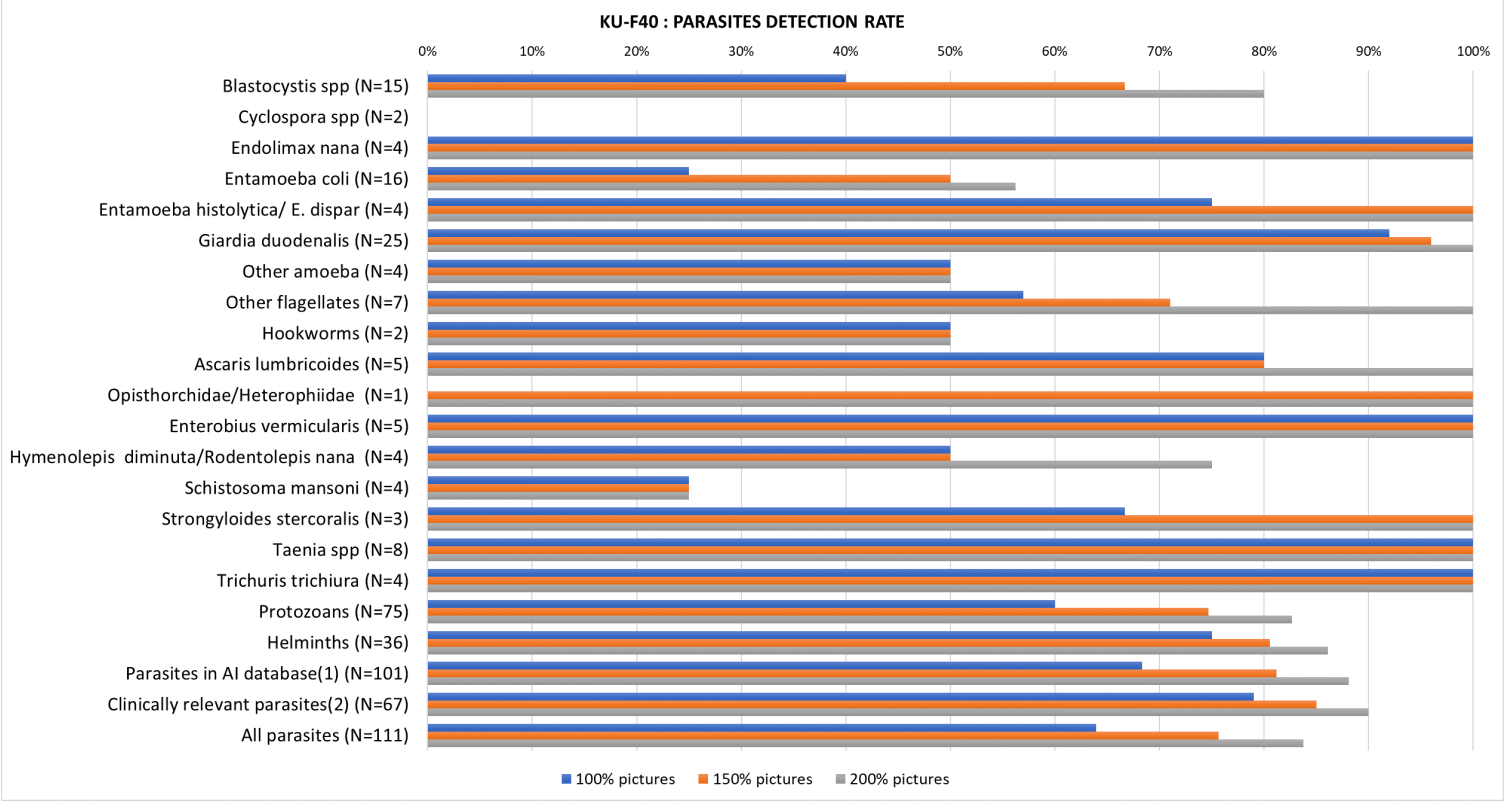
Changes in detection rate when increasing the number of pictures taken. (i) The following parasites are missing from the database: *Cyclospora* spp., *Rodentolepis nana*, *Hymenolepis diminuta,* and *Schistosoma mansoni*. (ii) We excluded *Entamoeba coli*, *Endolimax nana*, *Iodamoeba buetschlii*, *Entamoeba hartmanni*, *Chilomastix mesnilii*, *Dientamoeba fragilis,* and *Blastocystis hominis*.

All the 80 positive samples, with a total of 111 parasites, were re-analyzed with a 150% and 200% increase in the number of images, respectively. We noted an improvement in detection rate for most of the parasites studied, except for *Cyclospora* spp., hookworms, *Schistosoma mansoni,* and for amoeba absent from the AI database. Changes in the detection for each parasite are shown in Fig. 4. Overall, the detection rate of all parasites improved from 64% (71/111) to 84% (93/111) with the standard settings and the 200% increase in images, respectively. When considering only parasites present in the database, this rate increased from 68% (69/101) to 88% (89/101), respectively.

With the 200% increase in images, 18 parasites remained undetected by the KU-F40. After reviewing all the pictures taken, we found that 8 out of those 18 parasites were photographed but not detected by the AI: *Entamoeba coli* (3/16), *Entamoeba hartmanni* (1/2), *Blastocystis hominis* (1/15)*, Cyclospora spp*. (1/2), and *Schistosoma mansoni* (2/4). The 10 other parasites missed by the camera included *Entamoeba coli* (4/16), *Blastocystis hominis* (2/15), hookworm (1/2), *Rodentolepis nana* (1/3), *Cyclospora* spp. (1/2), and *Schistosoma mansoni* (*N* = 1). The sensitivity of the camera was then 91% (101/111) for all parasites, and 94% (77/82) for clinically relevant parasites.

#### Correct identification rate

The identification rate was calculated among parasites present in the database and automatically identifiable with the AI. Among the 111 parasites included, 88 met these criteria and 82 were correctly identified, yielding a correct identification rate of 93%. Among the misidentified parasites, we found *Entamoeba coli* (identification rate of 44%), identified as *Blastocystis* one time and as *E. histolytica/E. dispar* 4 out of 9 cases, and *Blastocystis hominis* (identification rate of 92%) identified as *G. duodenalis* in 1 out of 12 cases. Iodine treatment was performed on all samples harboring amoeba species, but it did not alter the identification results.

### Turnaround time (TAT)

Out of the three batches of 20 samples analyzed, we measured a mean TAT of 3.75 min per sample for KU-F40 and 5.20 min for manual microscopy. Regarding the effective manual working time for the technologist, it was 0.55 min per sample with the KU-F40, and 5.20 min for manual microscopy, resulting in a time saving of 1 h and 35 min for a series of 20 samples using KU-F40 compared with standard microscopy of a direct wet mount.

## DISCUSSION

The automation of microscopy, as proposed by the KU-F40 system, presents attractive advantages: standardization of sample preparation, photographic documentation of stool appearance, storage of images for remote reviews.

Regarding external quality control samples, the KU-F40 correctly detected 10 out of 11 parasites present in the eight tested samples. The missed parasite (*Trichuris trichiura*) was present, but Sciensano specified that it contained a very low burden and was also missed by 11% of participating Belgian laboratories (11/101). Standard microscopy allowed detection of this parasite.

Our comparative study highlights the inferiority of the KU-F40 compared with manual microscopy. With default settings, a sensitivity of 86% appears clearly insufficient if the KU-F40 is used as a screening tool with automatic validation of negative samples. However, it is precisely in this configuration that the system would have the most impact on workload. It reduces technical time by a factor of 10 in our study. In our laboratory, this corresponds to an average working time savings of 1 h and 45 min per week. Given the shortage of well-trained personnel and the large number of samples received, our current analysis time per sample is very short. As a result, the time savings could be even greater in other laboratories. Moreover, most laboratories use concentration techniques before microscopic analysis. While these techniques increase the TAT compared to an automated method like the KU-F40, the difference in sensitivity would be even more significant.

The lack of sensitivity could primarily be attributed to the absence of certain parasites in the AI database. To be detected and identified, a parasite must be recognized by the AI analyzing the images. However, at the time of this study, key parasites were missing from the database, leading to their systematic non-detection or, in the best-case scenario, misidentification, i.e. *Cyclospora* spp., a worldwide distributed pathogen and *Schistosoma* spp. whose significance continues to grow given the growing immigrant population in Europe. The system is expected to evolve through version updates, which will influence its current performance.

Additionally, the sample preparation technique is questionable. The KU-F40 dilutes stool samples during homogenization and performs filtration without concentration. While filtration alone is mentioned in some guidelines, it is also recommended that the entire filtrate should be examined, requiring multiple slides (14). However, the KU-F40 analyzes only one slide, which is not fully examined.

Finally, the absence of a 10× objective, which normally allows for rapid screening of the entire slide, is a key missing element. This may explain the absence of certain helminth eggs, yet easily found at 10× magnification with the standard microscopy, such as eggs of *Rodentolepis nana*, of *Schistosoma mansoni*, or eggs of hookworms.

False positives are frequently encountered with the KU-F40, with nearly one in ten samples falsely flagged as positive for artefactual elements identified as *Dibothriocephalus* (and related genus) or *Blastocystis*. However, reviewing these false positives is not a major issue and is relatively quick, as the flagged images appear first in the result report.

Overall, 93% (82/88) of the parasites present in the database were correctly identified. Identification errors mainly involved *Entamoeba coli* identified as *E. histolytica/dispar*. Unfortunately, the quality of KU-F40 images did not allow for proper differentiation of these species from other *Entamoeba* spp., even after iodine staining. In practice, these samples should be reviewed using manual microscopy for accurate identification. For all other parasites, image quality was inferior to manual microscopy, but still sufficient for easy identification.

While the contamination study for *Giardia duodenalis* was conclusive, the contamination issue observed with the *Taenia* egg in the comparative study is particularly problematic. Our study does not appear to be robust enough to fully assess the risk of contamination. In our data set, we observed one contamination event among 22 instances where a negative sample immediately followed a highly positive sample within the same channel (4.5%). In contrast, no contamination was observed among 158 cases where a negative sample followed another negative sample. Given the small sample size, further investigation with a larger data set is warranted to more robustly assess this risk. As recommended by the manufacturer, routine use of the daily cleaning solution and weekly application of the more concentrated solution did not prevent this incident. In routine practice, this could lead to diagnostic and treatment errors. A program should be developed in the KU-F40 application to analyze all fields across the four slides using a single cleaning solution. This would help to exclude contamination, for example, by implementing a system-wide analysis at the end of each working day.

Finally, we observed that increasing the number of captured images by 200% improved detection rates for all parasites in the database, except hookworms. However, this parasite was only present in two samples. The overall parasite detection rate increased to 84% for all tested parasites and 88% for those included in the database. After reviewing all images for each sample, 91% of parasites (101/111) were visible in the captured images (including both automatically detected and undetected parasites). Among the parasites included in the database, the KU platform demonstrated notably low sensitivity for *Blastocystis* spp. and *Entamoeba coli* (either missed by the camera or not recognized by the AI), which does not support the use of this solution for reliable diagnosis of these organisms.

To our knowledge, this study is the second to evaluate the performance of the KU-F40 microscopy module. The first study was conducted in Guangxi Province, China (15). The authors tested 1,030 stool samples, of which 236 (23%) were positive, although almost exclusively for *Opisthorchidae/Heterophiidae* eggs (232/236). The sensitivity and specificity of the parasite detection in the standard mode of the KU-F40 were evaluated at 83.1% and 100%, respectively. We therefore note a major difference in performance, particularly in specificity, compared with this first study. However, it is important to note that the vast majority (98%) of tested parasites in that study belonged to the same genus, which may account for the marked difference in performance compared to our study. Differences in the epidemiology of IPI between countries should lead to a systematic evaluation of the performance of this type of automated system. A second flotation-sedimentation mode, which was not tested in our study, showed lower performance, with a sensitivity of 52.1% and a specificity of 97.7%. The good performance of the antigen tests available on the KU-F40 has already been published (16). Prospective studies should be conducted to compare manual microscopy, automated microscopy, and multiplex PCR panels to accurately assess the performance of these different methods and determine the most appropriate type of automation.

Several other commercially available systems support the examination of stool samples for ova and parasites. Among them, Techcyte (Techcyte, Inc., Linden, UT, USA) demonstrates excellent performance for protozoan detection, with sensitivity and specificity above 98%, and a limit of detection five times lower than that of manual microscopy (17, 18).

The AVE-562 analyzer (AVE Science & Technology Co., Hunan, China), specifically evaluated for detecting *Opisthorchidae* and *Heterophyidae* eggs, showed low sensitivity (36.4%) but perfect specificity (19).

The Oriental Model FA280 (Orienter, Chengdu, Sichuan, China), when compared with manual microscopy using the formalin-ether concentration technique (FECT), exhibited good sensitivity (94%). While initial agreement between FECT and the FA280 identification was only fair, accuracy reached 100% following manual review of FA280-generated images (20).

The sediMAX I and sediMAX II systems (Menarini Diagnostics, Florence, Italy) also showed excellent sensitivity and specificity for detecting various protozoa and helminth eggs. These platforms offer automated image acquisition and storage; however, images still require manual review for parasite identification (21, 22).

Other systems with similar functionalities exist but are primarily designed for veterinary use, such as the HESKA Element AIM (Antech Diagnostics Inc., Vermont, Australia) and FECPAKG2 (Techion, Mosgiel, New Zealand).

This study has several limitations. First, our sample set is not representative. The number of certain parasites tested was low, as they are rarely encountered in our country, which limits the ability of our study to fully assess the performance for each parasite tested. Additionally, a key and frequently observed parasite, *Cryptosporidium* spp., was absent. Although we collected *Cryptosporidium*-positive samples, this parasite was primarily detected by rapid antigen testing rather than manual microscopy. Therefore, we chose not to include these samples in the present study. However, it would be interesting to validate the KU-F40 *Cryptosporidium* antigen test, not available at the time of this study. Sensitivity could be significantly improved by testing together the microscopic module alongside antigen testing for *Giardia* and *Cryptosporidium*, two of the most commonly encountered parasites in our country. Another limitation is that only positive samples were reanalyzed with an increased number of images. This allows us to assess the impact on detection rates but not on specificity. The limitation concerning our contamination study has already been discussed above. We underline that a larger number of replicates, including highly loaded samples with different parasites, would be necessary to assess this risk. Finally, in this study, we used the default threshold values established by the manufacturer for each parasite (see the supplementary material at https://doi.org/10.5281/zenodo.18268350). Although not recommended by the manufacturer, these values can be manually adjusted to affect the sensitivity and specificity of the system on a parasite-by-parasite basis. This option was not explored in the present study.

In conclusion, the KU-F40 offers an innovative approach and provides welcome automation in the diagnosis of intestinal parasitic infections with the advantage that it remains microscopy-based. Currently, its performance does not allow for its use as a screening tool with automatic validation of negative results. However, it could be employed as a first-line tool with manual review of all images. With default settings, KU-F40 showed particularly low sensitivity for *Entamoeba coli* and *Blastocystis spp*., which are among the most frequently encountered intestinal protozoa. Some key missing features could enhance its performance, including the addition of a 10× magnification objective, the ability to rapidly detect system-wide contamination, and the inclusion of additional parasites currently absent from the database, adapted to the epidemiology of other continents.

## Data Availability

The data that support the findings of this study are available in the article. The detailed clinical/biological data of patients are not publicly available due to privacy or ethical restrictions.
